# Effect of Fiber Content on the Preparation and Mechanical Properties of 3D Printed Short Carbon Fiber Reinforced PA Composites

**DOI:** 10.3390/polym17050671

**Published:** 2025-03-02

**Authors:** Yesong Wang, Feilong Li, Zixuan Sun, Chenyu Gu, Kunkun Fu, Xiangming Zhao

**Affiliations:** 1 School of Mechanical Engineering, Jiangsu University of Science and Technology, Zhenjiang 212000, China; 231210201310@stu.just.edu.cn (F.L.); 221210201408@stu.just.edu.cn (Z.S.); 241210201206@stu.just.edu.cn (C.G.); 2 School of Aerospace Engineering and Applied Mechanics, Tongji University, Shanghai 200092, China; 3 Suzhou FusRock New Materials Co., Ltd., Suzhou 215500, China; walter.zhao@fusrock.com

**Keywords:** additive manufacturing, polymer composites, mechanical properties, fused deposition modeling

## Abstract

3D-printed short-carbon-fiber-reinforced thermoplastic composites have attracted significant attention from both the academic and industrial communities due to their remarkable advantages such as lightweight, high strength, and recyclability. However, in most of the current 3D-printing-related nylon composites, the content of short carbon fibers is generally low, and the influence laws of short carbon fibers on the mechanical properties of the composites have not been fully explored. This paper focuses on short-carbon-fiber-reinforced nylon (SCF/PA) composites with short-carbon-fiber contents of 15 wt%, 25 wt%, and 35 wt%, respectively. It studies in depth their mechanical properties and related characteristics. The research results show that with the increase in the short-carbon-fiber content, the melt flow rate of the SCF/PA composites shows a downward trend. In terms of mechanical properties, when the short-carbon-fiber content is 25 wt%, the tensile strength and flexural strength of the composite reach their maximum values, which are 101.43 MPa and 173.16 MPa, respectively. Compared with pure nylon, the improvement ranges are 17.01% and 21.4%, respectively. When the short-carbon-fiber content is 35 wt%, the impact resistance of the material reaches its optimal value, which is 6.02 KJ/m^2^, an increase of 38.1% compared with pure nylon. At the same time, when the short-carbon-fiber content is 35 wt%, the thermal deformation temperature of the material also shows a certain degree of slight increase. In summary, the research results of this paper will provide more abundant and detailed experimental data support for 3D-printed short-carbon-fiber-reinforced nylon composites in various different application scenarios, facilitating further exploration and application in related fields.

## 1. Introduction

Additive manufacturing (AM) technology [1], also known as 3D printing technology, uses materials to produce solid parts in a layer-by-layer manner. It represents a modern approach in manufacturing and processing technology. Due to its ability to process complex shapes and hard-to-machine parts, it is widely applied in aerospace and other industries. 3D printing technology can be classified into different types based on the products or materials produced. These include fused deposition modeling (FDM), photosensitive resin selective curing (SLA), powder material selective laser sintering (SLS), and other technologies [2,3,4]. Among them, the fused deposition technology is more popular. It has advantages such as a wider range of available materials, low cost, and a high material utilization rate.

Among the materials used in fused-deposition technology, unreinforced materials like ABS, PA6, PP, PLA, and PC exhibit significantly lower mechanical properties compared to fiber-reinforced composites. These unreinforced materials are mainly used for manufacturing conceptual models rather than being employed as load-bearing or functional components [5] in industries such as automotive, aerospace, and medical devices. This situation has significantly restricted the development of fused-deposition technology. Therefore, it is necessary to enhance the mechanical properties of these resin materials so that the FDM technology can have greater opportunities and better prospects for development in automotive and other fields.

Carbon fiber, boasting high strength, a high modulus, high-temperature resistance, corrosion resistance, and other outstanding properties, serves as a crucial reinforcing material for structural applications. It is extensively applied in industries such as aerospace, automotive, high-end sports equipment, and high-speed rail [6,7,8,9]. Short-carbon-fiber-reinforced composites, characterized by their suitability for structures with more complex geometries and low cost, have attracted significant attention.

Numerous experts and scholars have conducted in-depth research on short-carbon-fiber-reinforced composites using FDM technology [10,11,12]. In addition, a certain amount of headway has been achieved in the study of how the content of short carbon fibers impacts the mechanical properties of composites. Studies have shown that adding different amounts of short carbon fibers to materials such as ABS, PP, and PEEK can significantly improve the mechanical properties of the composites [13,14,15,16].

As an engineering plastic, nylon can form a lot of hydrogen bonds between its molecules [17], with good mechanical properties and thermal stability [18], so the short carbon fiber as a composite reinforcing body, nylon as a matrix formed by the short-carbon-fiber-reinforced nylon composites have the advantages of low density, high abrasion resistance, and light weight as well as high mechanical properties.

Many experts and scholars have studied the influence of short-carbon-fiber-reinforced composites on mechanical properties through 3D printing technology from different angles such as the construction direction, grating angle, layer height, filling pattern, etc. [19,20]. Current research on the effects of short carbon fiber content on the mechanical properties of short-carbon-fiber-nylon composites in terms of 3D printing stays at the stage of lower content of short carbon fibers [21,22]. For example, Elena Verdejo de Toro et al. [23] studied six 20 wt% short carbon fiber nylon composites by the FDM molding process and polymer injection (PIM) molding process for comparison. The results show that both the 3D printing and PIM processes resulted in fiber fracture, shortening the fibers by up to 24%. Tensile tests of the printed samples showed poorer results than the PIM samples, and compression tests showed similar results measured by the two processes. Md Niamul Islam et al. [22] also prepared 20 wt% short-carbon-fiber-nylon composites, and the results were similar to those of Elena Verdejo de Toro et al. [23]. The samples produced by Elena Verdejo de Toro et al. [23] had a tensile strength of 52 MPa, and their tensile strength was enhanced to 78.91 MPa, while the tensile properties were significantly improved. Constantina et al. [24] explored the effect of the short-carbon-fiber content and grating angle on the tensile properties of short-carbon-fiber-reinforced composites, and concluded that 10% short-carbon-fiber composites at a 0^0^ °grating angle had the best tensile properties, and the finite element analysis was used to apply these composites to the medical field. Flaviana Calignano et al. [25] used 20 wt% short-carbon-fiber-nylon composites to study the effect of the construction direction and filler percentage on the hardness, tensile properties and elasticity, and found that the samples constructed in the XY plane in the construction direction had higher hardness than those constructed in the XZ plane, and that tensile results of specimens constructed in the XZ direction had a higher Young’s modulus. The specimens had a higher Young’s modulus and stress at break, and it was found that the mechanical properties were independent of the filling factor. Nevin Gamze Karsli et al. [26] investigated the tensile strength, modulus and hardness values of PA6 reinforced with carbon fibers with a different content (up to 20 wt%) and length of short carbon fibers. The fiber volume fraction is a crucial driving factor for the mechanical properties of composites and directly affects the mechanical properties of short-carbon-fiber composites. Therefore, it is of great significance to study SCF/PA composites, including those with a high content of short carbon fibers. Dakota R Hetrick et al. [27] studied the fitting effects of the Voigt, Reuss, and Halpin–Tsai models on the mechanical property tests of composites with four different short-carbon-fiber volume fractions of 13.8%, 27.5%, 41.3%, and 55%. The Voigt model provides an excellent fit for the longitudinal tensile strength and a reasonable fit for the longitudinal modulus. In the transverse direction, although the Reuss model cannot accurately reflect the trend of the transverse modulus, the Halpin–Tsai model provides a more reasonable fit because it incorporates more experimental parameters. Through the above research, it has been found that the influence of the short-carbon-fiber volume fraction on the mechanical properties of composites has not been fully studied and analyzed, and there is a lack of analysis of the flow characteristics of short-carbon-fiber-reinforced nylon composites with a high content of short carbon fibers.

In this paper, three kinds of short-carbon-fiber-reinforced nylon composites with mass fractions of 15 wt%, 25 wt%, and 35 wt% were prepared by mixing a certain proportion of short carbon fibers and nylon through a twin-screw extruder. The composite filaments were extruded by a single-screw extruder, and the pure nylon resin was used as a control test. Based on 3D printing, tensile specimens, flexural specimens, impact specimens, and thermal-deformation specimens were printed to analyze and study the influence rules of the short-carbon-fiber content on mechanical properties and flow characteristics. Then, the cross-sectional morphology of the printed specimens after the tensile test was analyzed using a scanning electron microscope. The effect of different contents of short carbon fibers on the mechanical properties of SCF/PA composites and the fracture mechanism was analyzed from the microstructure.

## 2. Materials and Methods

### 2.1. Materials

The resin in this experiment is nylon produced by the Shandong Guangyin New Materials Co.,Ltd., and the short carbon fibers are selected from the products of the Mitsubishi chemical corporation of Japan. The specific performance indices are shown in Table 1.

### 2.2. Experimental Equipment

The main purpose of this paper is to analyze and compare the effects of different contents of short carbon fibers on the mechanical properties of SCF/PA composites. The samples were prepared by a Bamboo P1S model printer, The equipment is produced by Shenzhen Bambu Lab Co., Ltd., Shenzhen, China, the tensile and flexural strengths were tested by an ETM104B universal testing machine produced by the Shenzhen Wance Testing machine Co., Ltd., Shenzhen, China; the impact strength was tested by PIT501 J impact testing equipment produced by the Shenzhen Wance Testing machine Co., Ltd., Shenzhen, China, and the thermal deformation temperature was tested by a HVT302A Microcomputer-controlled Thermal Deformation Vicat Softening Point Testing Machine, The equipment is produced by the Shenzhen Wance Testing machine Co., Ltd. Shenzhen, China. The interface was scanned by a ZEISS Sigma 360 scanning electron microscope, Baden-Württemberg, Germany. The melt flow rate test was performed using the XNR-400A type melt flow rate meter produced by the Chengde Jinhe Instrument Manufacturing Co., Ltd., Chengde, China.

## 3. Experimental Methods

### 3.1. Preparation and Printing of SCF/PA Composites

**Figure 1:**
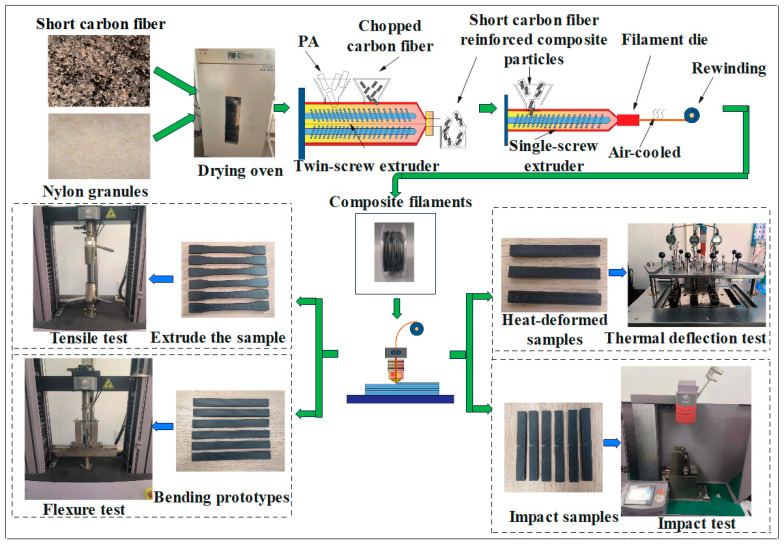
Diagram of the experimental program.

Firstly, the short carbon fiber and nylon resin were dehydrated in a 120 °C drying oven for 6 h. The short-carbon-fiber-reinforced nylon (SCF/PA) composite particles were prepared by fully mixing nylon and short carbon fibers in a specific ratio using a twin-screw extruder. Then, the SCF/PA composite particles were squeezed into a single-screw extruder. The composite material was melted into a viscous liquid at a high temperature and shaped into filaments. Finally, SCF/PA composite filaments with a short-carbon-fiber content of 15 wt%, 25 wt%, and 35 wt% were prepared. The prepared composite filaments were printed with a 3D printer to test the mechanical properties of the test samples. The experimental scheme is shown in Figure 1.

### 3.2. Properties of Composite Materials

#### 3.2.1. The Content of Single Filament Performance Test

In the production process, the monofilament diameter of SCF/PA composites with different contents will be monitored in real time by the diameter measuring instrument. If it is found that the diameter data are too large or too small, the equipment will make corresponding adjustments. Therefore, the monofilament is basically produced with the monofilament diameter in accordance with the ISO 5425: 2023 standard [28]. The subsequent test measuring instrument randomly collects five real-time measured point diameter data by sampling, and takes the average value of the five-point diameter data as the final result.

The melt flowability of composites is characterized by the melt flow rate (MFR). In accordance with the ISO 1133-1:2022 standard [29], the XNR-400A apparatus was used to test the effect of the short-carbon-fiber content on the flow properties of SCF/PA composites. The experimental preparations were carried out by placing short-carbon-fiber-reinforced nylon pellets of different contents in a drying oven at 100 °C for more than 4 h. The test temperature was 300 °C and the load weight was 2.16 kg. And the final results were recorded as the melt mass every 10 min.

According to the ISO 5425:2023 standard [28], five monofilaments with a fixture spacing of 350 mm were taken for the monofilament tensile test of the prepared SCF/PA composites with different contents. The tensile speed was 50 mm/min, and the average of the five data was taken as the result.

#### 3.2.2. The Content of Single Filament Performance Test

**Figure 2:**
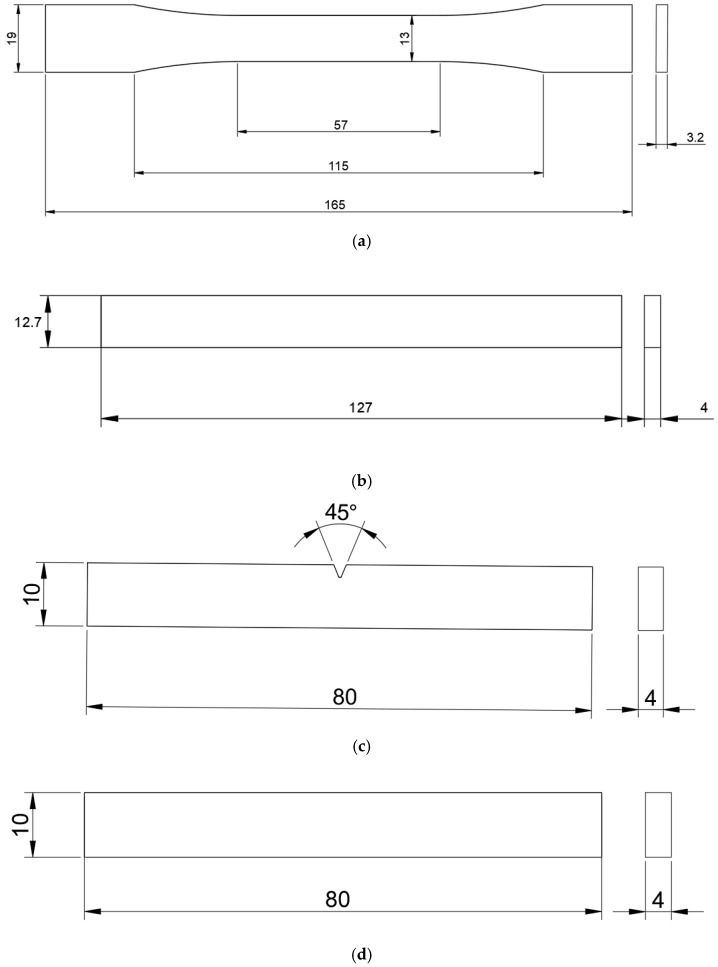
Printed dimensions of test samples. (**a**) Tensile sample; (**b**) flexural sample; (**c**) Impact sample; (**d**) Thermal deformation sample.

First of all, the test samples are designed by computer-aided design software UG and saved in the STL file format, then uploaded to a 3D printer slicing software Bambu studio, the version number is 01.10.01.50, and then sliced by the slicing software of the 3D printer to print out the tensile samples, flexural samples, impact samples, and thermal deformation samples. Tensile samples are printed according to the ASTM D638I standard [30] design, as shown in Figure 2a. Flexural samples are printed according to the ASTM D790 standard [31] design, and the size of the design is shown in Figure 2b. The impact samples are designed according to the ISO 179-1 standard [32], and the pendulum is set to 150° and released quickly along the direction opposite to the notch of the impact specimen. The specific dimensions are shown in Figure 2c. The heat deformation sample is designed according to the ISO 75-1-2013 standard [32,33] size. The design test span is 64 ± 1 mm, and 0.45 MPa flexural stress is applied. The heating device will rise uniformly at a heating rate of 120 °C/h. The specific size is shown in Figure 2d.

The printing parameters are shown in Table 2; tensile samples, flexural samples, impact samples of each group of six prints, thermal deformation according to the relevant standards to print three, due to 3D printing taking into account the anisotropy of the material; the provisions of a uniform print orientation ±45° direction; the test is taken after the average value of the data of each group to represent the performance parameters.

The tensile strength and tensile modulus are calculated by the following Equations (1) and (2).

(1) $$\sigma =\frac{F}{A}$$

(2) $$E=\frac{FL}{A\left(\Delta L\right)}$$

In Equations (1) and (2), *σ* is the tensile stress, *F* is the tensile force, *A* is the tensile force area, *E* is the tensile modulus, *L* is the standard spacing, and ∆*L* is the increment of standard gauge spacing.

The flexural strength and flexural modulus are calculated by the following Equations (3) and (4)

(3) $$\sigma_{f}=\frac{3PL}{2bd^{2}}$$

(4) $$E_{B}=\frac{L^{3}m}{4bd^{3}}$$

In Equations (3) and (4), $\sigma_{f}$ is the flexural stress, Unit MPa, *P* is the given load on the load-displacement curve, *b* is the width of the sample, d is the thickness of the sample, *L* is the span length, $E_{B}$ is the flexural modulus of elasticity, *L* is the span length, and m is the slope of the initial linear segment of the load-displacement curve.

The notched specimen simply supported the beam impact strength $a_{cN}$ calculated in accordance with Formula (5), notched for type A, unit kilojoules per square meter (kJ/m^2^);

(5) $$a_{cN}=\frac{E_{c}}{h\cdot b_{N}}\times 10^{3}$$

In the formula, $E_{C}$ is the energy absorbed when the modified specimen is destroyed, unit joule (J), *h*—sample thickness, unit millimeter (mm), $b_{N}$ sample residual width, unit millimeter (mm).

### 3.3. Sectional Morphology Analysis of Composites

A scanning electron microscope (SEM) was used to observe the fracture interface of the tensile samples and analyze the section, and the damage mechanism of the samples was further analyzed and interpreted from a microscopic point of view on the section morphology. This equipment was manufactured in the state of Baden-Württemberg, Germany.

## 4. Results and Analysis

### 4.1. Monofilament Performance Tests

#### 4.1.1. Monofilament Diameter

As shown in Table 3, it can be seen from the test results that from 0 wt% SCF/PA to 35 wt% SCF/PA, the diameter is uniformly distributed. According to the ISO5425:2023 wire diameter standard, the diameter is required to be 1.75 ± 0.03 mm. The test results comply with the wire diameter standard, which provides a basis for subsequent research.

#### 4.1.2. Melt Flow Rate Properties

**Figure 3:**
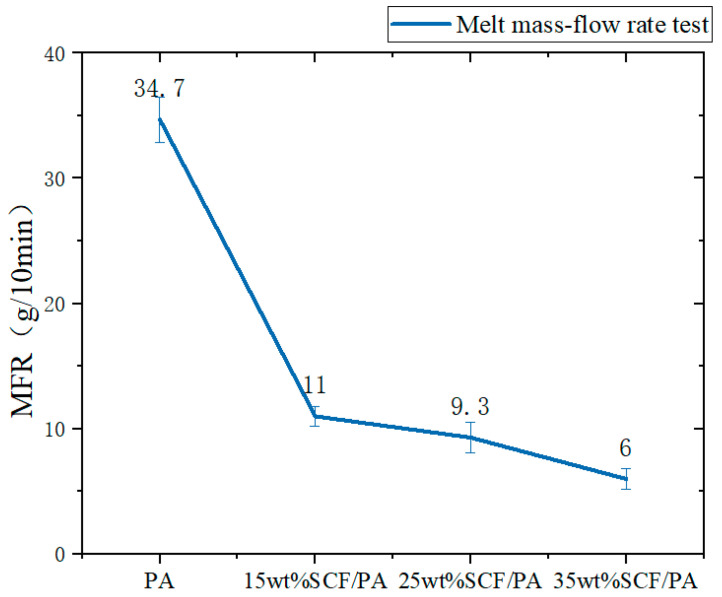
Effect of short-carbon-fiber content on melt flow rate.

The effect of the short-carbon-fiber content on the melt flow rate is shown in Figure 3. From this figure, it can be observed that the melt flow rate of the composites shows a decreasing pattern with the increase in the short-carbon-fiber content. This is because the addition of short carbon fibers increases the viscosity of the melt, and the strong interaction and friction between the short carbon fibers and the nylon hinders the flow and slip of the polymer molecules, so that the melt needs to overcome a greater resistance during the flow process, which leads to a decrease in the melt flow rate. Additionally, the addition of short carbon fibers may affect the regular arrangement and movement of the nylon molecular chain and further restrict the fluidity of the melt, thus increasing the resistance to flow. The higher the added SCF content is, the greater is the flow resistance.

#### 4.1.3. Monofilament Tensile Properties

**Figure 4:**
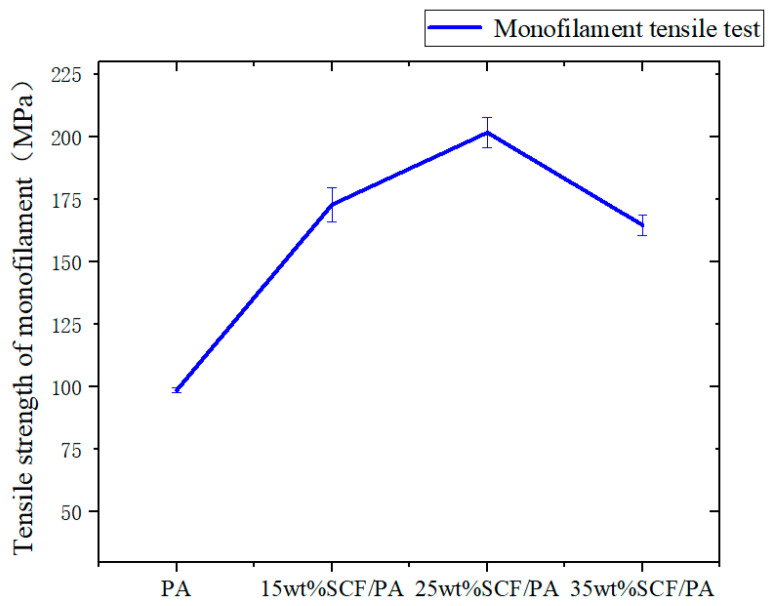
Monofilament tensile data.

The results of the monofilament tensile strength test with different short-carbon-fiber contents are shown in Table 4. From Figure 4, it can be seen that the tensile strength of the monofilament gradually increases with the increase in the short-carbon-fiber content from 0 wt% SCF/PA to 25 wt% SCF/PA, and reaches the maximum at 25 wt% SCF/PA, while the maximum monofilament tensile force is 201.75 MPa. However, the tensile strength of 35 wt% SCF/PA decreased significantly, indicating that the increase in short-carbon-fiber content could not provide higher strength for the composite.

### 4.2. Printer Performance Tests

#### 4.2.1. Tensile Properties

**Figure 5:**
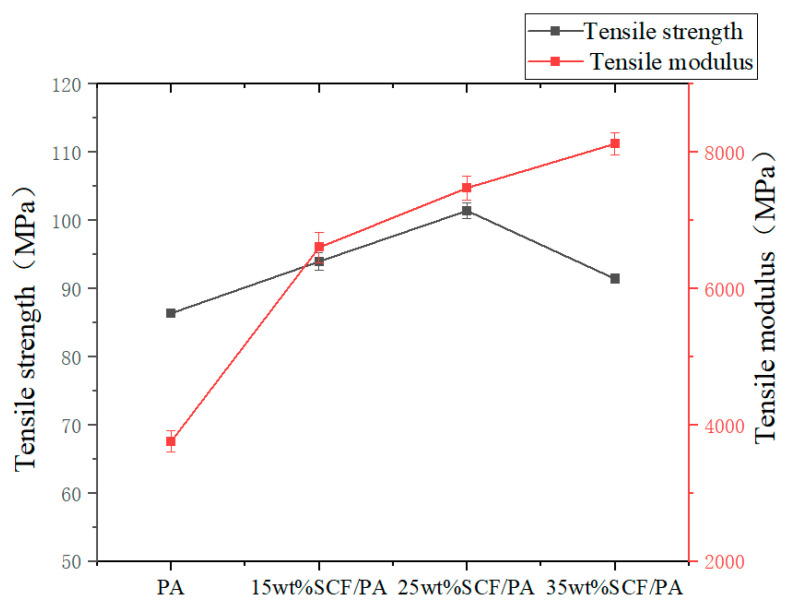
Effect of short carbon fibers on tensile strength and tensile modulus.

The effect of the short-carbon-fiber content on the tensile strength and tensile modulus is shown in Figure 5. Firstly, it can be seen from the figure that the overall tensile strength of the added short carbon fibers is significantly improved relative to that of pure nylon. Specifically, when the short-carbon-fiber content increases from 0 wt% to 25 wt%, the tensile strength gradually increases and reaches the maximum tensile strength of 101.43 MPa at 25 wt% SCF/PA composites, which is 17.01% higher than that of 86.63 MPa of pure nylon, and the short carbon fiber content decreases at 35 wt%, but it is also higher than that of pure nylon. This is basically consistent with the test monofilament strength performance law. Secondly, the tensile modulus was observed; with the increase in the short-carbon-fiber content, the tensile modulus showed a gradual increasing trend, and the short-carbon-fiber content of 35 wt% reached the maximum of 8124.84 MPa, which was 125.37% higher than that of pure nylon.

Because the short carbon fiber is added to the nylon as a reinforcing material, the short carbon fiber can be fully wrapped by the PA, and the uniform wrapping of the short carbon fiber means that the two have a large enough contact area. When the tensile strength test is carried out, the PA is subjected to the force at the same time. In time, it will be transmitted to the closely contacted short carbon fiber to realize the transmission of force, which is reflected in the improvement in the tensile strength from a macro perspective.

When the carbon fiber content is 25 wt%, the tensile strength reaches its maximum value. At this time, a large number of short carbon fibers are uniformly distributed in the nylon, with almost no fiber aggregation, and there is some nylon bonded to the surface of the short carbon fibers, indicating that the interfacial bonding force between the short carbon fibers and nylon is strong. This strong interfacial bonding force and the uniform distribution of short carbon fibers enable the tensile strength of the SCF/PA composite to reach its maximum. A decline occurs at 35 wt%, indicating that an increase in the short-carbon-fiber content will no longer enhance the tensile strength of the SCF/PA composite material. This is because the excessive addition of short carbon fibers cannot be fully wrapped in PA, resulting in the aggregation of short carbon fibers and uneven distribution. When the sample is subjected to a tensile load, the force can only be transmitted to the short carbon fibers in contact with PA. Due to the aggregation of short carbon fibers, there is only a small friction between the internal short carbon fibers, which will inevitably lead to a decrease in tensile strength. Therefore, the tensile strength decreases when the content of short carbon fibers is 35 wt%, but it also improves compared with pure nylon. The tensile modulus is still increasing when the content of short carbon fibers is 35 wt%, which shows that although there is a decrease in tensile strength, the carbon fibers themselves have a high modulus, and the short carbon fibers play a dominant role in the resistance to deformation and improve the rigidity of the overall material; this makes the composite material more prone to fracture but the ability to resist deformation before fracture is improved.

From the tensile performance data in Table 5, we can observe the tensile strain of the tensile sample. The influence of the short-carbon-fiber content on the ductility of different parts of the sample can be seen. The ductility gradually decreases as the short-carbon-fiber content increases from 0 wt% to 25 wt%. When the short-carbon-fiber content is 35 wt%, the ductility is basically the same as that at 25 wt%. The average ductility is the largest at 0 wt% SCF/PA, which is 5.35%. When the short-carbon-fiber content is 25 wt%, the ductility is the smallest, which is 2.16%.

#### 4.2.2. Flexural Properties

**Figure 6:**
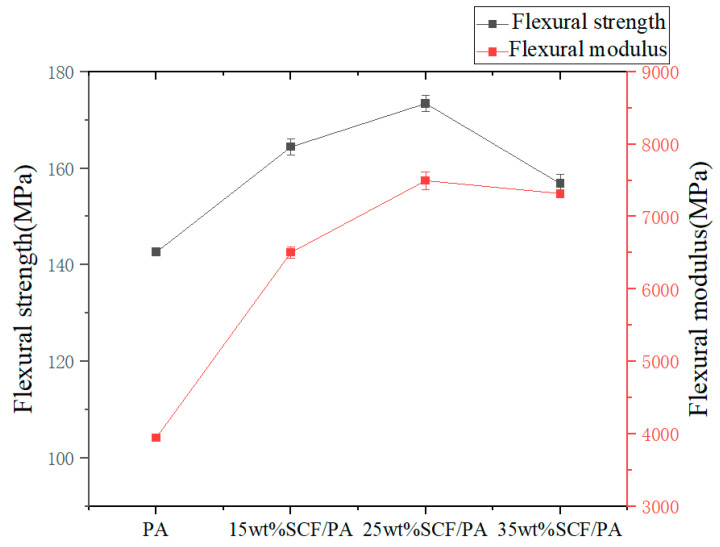
Effect of short carbon fibers on flexural strength and flexural modulus.

The flexural strength and flexural modulus of the composites were measured by three-point flexural experiments, as shown in Figure 6, which presents the effect of the short-carbon-fiber content on the flexural strength and flexural modulus. From the figure, we can see that the overall enhancement effect of short carbon fibers on the composite material is obvious, showing an overall trend of first rising and then declining with the increase in the short-carbon-fiber content. Specifically, when the short-carbon-fiber content is in the range of 0 wt% to 25 wt%, the flexural strength gradually increases. It reaches a maximum of 173.16 MPa at 25 wt% of the short-carbon-fiber content, which is 21.4% higher than that of pure nylon. And when the content is 35 wt%, it is in a declining stage, and the flexural strength is the lowest among the short-carbon-fiber addition cases, which is 156.82 MPa, 10% higher than that of pure nylon. The specific data are shown in Table 6: flexural properties data. In Figure 6, the effect of different short-carbon-fiber contents on the flexural modulus can be observed, showing the same trend as the flexural strength, i.e., showing an upward trend in the range of 0 wt% to 25 wt% and then beginning to decline. The flexural modulus reaches a maximum of 7546.99 MPa at 25 wt% of short carbon fibers, which is 91.2% higher than that of pure nylon.

#### 4.2.3. Impact Properties

**Figure 7:**
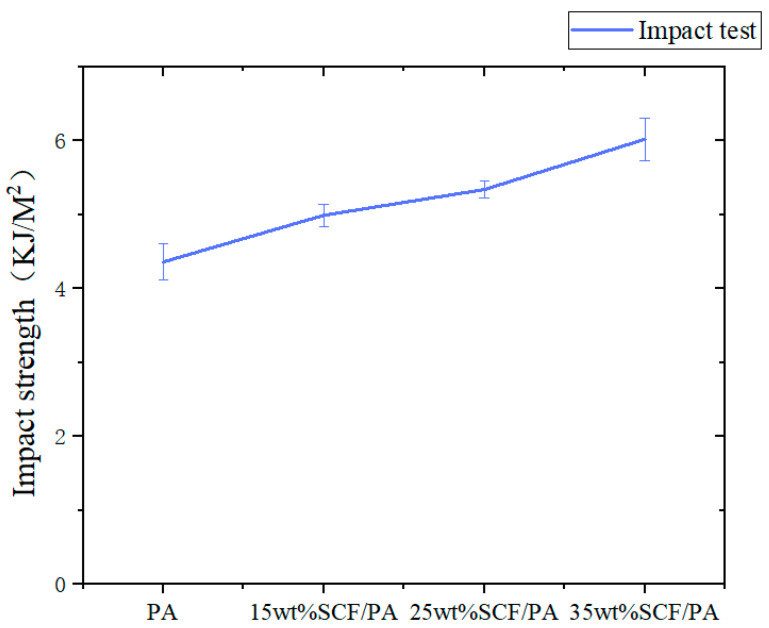
Effect of impact strength of short-carbon-fiber content.

The purpose of the impact test is to evaluate the ability of the material to absorb energy and resist the force generated by the external impact during the impact process. Table 7 shows the impact test data of short-carbon-fiber composite materials, and each group of data of this experiment is recorded. The effect of the short-carbon-fiber content on the impact strength is shown in Figure 7. The results show that the short-carbon-fiber nylon composite increases with the increase in the content, showing a positive correlation. Compared with pure nylon 4.36 KJ/m^2^, the impact strength of SCF/PA composites with the content of 35 wt% is 6.02 KJ/m^2^, which is increased by 38.1%. It can be seen that the increase in the short-carbon-fiber content improves the impact resistance of short-carbon-fiber composites. This is mainly due to the fact that the short carbon fiber and the resin rely on the interface between the two to achieve a load transfer, which is an important way of absorbing impact energy, and the short carbon fiber has a good strength and modulus. When the content of the short carbon fiber is low, the load transferred between the short carbon fiber and the resin is less and cannot absorb more impact energy. And short carbon fibers share a greater part of the impact energy and increase the impact resistance of SCF/PA composites, preventing the diffusion and propagation of cracks, thereby improving the overall impact resistance of the material.

#### 4.2.4. Heat Distortion Temperature Properties

**Figure 8:**
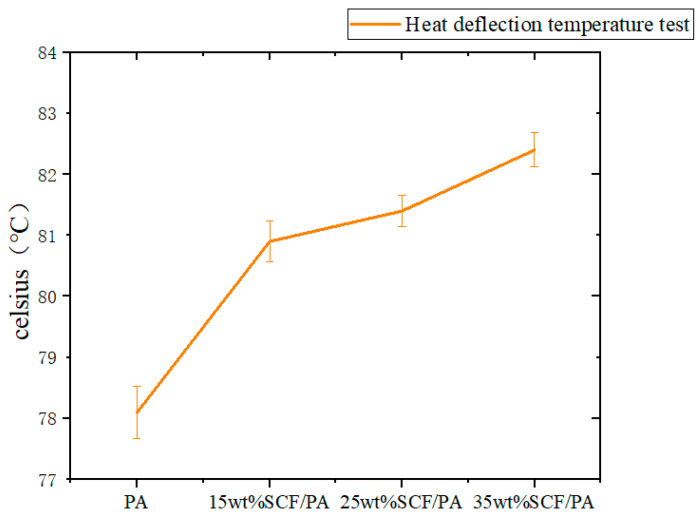
Effect of short-carbon-fiber content on heat distortion temperature.

Figure 8 shows the effect of the short-carbon-fiber content on the thermal deformation temperature, and the thermal deformation temperature and trend changes in these four materials are measured. From the overall trend, it can be seen that with the increase in the short-carbon-fiber content, the thermal deformation temperature will increase slightly. The 35 wt% SCF/PA composite material reaches the maximum thermal deformation temperature of 82.4 °C. This is because the addition of the short carbon fiber improves the thermal conductivity of the material. The more uniform heat conduction reduces the thermal stress inside the material, thus increasing the thermal deformation temperature to a certain extent. Also, the short carbon fiber has high stiffness and strength. When dispersed in nylon, it will limit the movement of the nylon molecular chain. This restriction makes it difficult for the molecular chain of the composite to undergo large displacement when heated, thereby increasing the thermal deformation temperature. Table 8 shows the specific test data of the thermal deformation temperature and all experimental data are recorded in it.

### 4.3. Sectional Observation and Fracture Mechanism Analysis of Sample Parts

**Figure 9:**
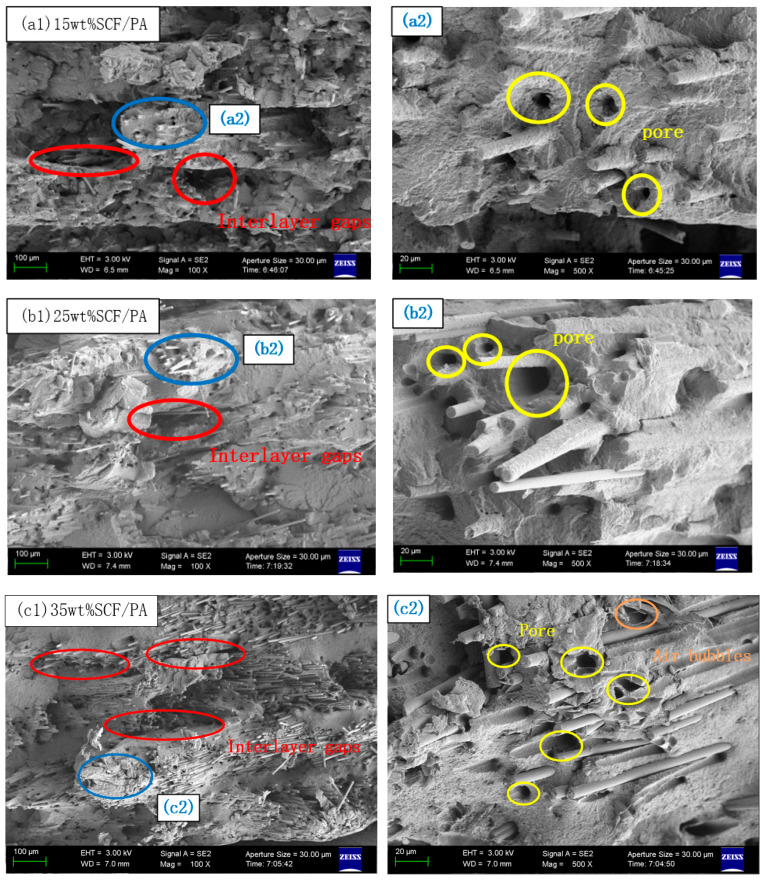
SEM of fracture surface of tensile samples with different contents of SCF/PA. (**a2**) 15wt% SCF/PA composite cross - section at 20 μm; (**b2**) 25wt% SCF/PA composite cross - section at 20 μm; (**c2**) 35wt% SCF/PA composite cross-section at 20 μm.

The fracture interfaces of SCF/PA composites with different contents after a tensile test were observed by a scanning electron microscope. The fusion infiltration of the short carbon fiber and nylon was explored from the microscopic point of view, and the fracture mechanism was further analyzed. As shown in Figure 9, the fracture surfaces of SCF/PA tensile samples with different contents were observed by SEM. It can be seen from Figure 9a1–c1 that with the increase in the carbon fiber content, the number of fibers on the fracture interface increases, and more fibers and pores are observed on the fracture surface. The pores circled by red circles are interlaminar pores. It can be seen from Figure 9a2–b2 that most of the short carbon fibers are uniformly wrapped by nylon resin at the interface. There is no large area of agglomeration between the fibers. The pores circled by yellow circles in the picture are the holes that are pulled out when the fibers are broken. When the short-carbon-fiber content is 15 wt% and 25 wt%, it can be seen that there is a certain amount of nylon matrix material attached to the surface of the pulled short carbon fiber. The surface is smoother at 35 wt%, indicating that the nylon effectively transmitted the tensile force to the carbon fiber itself when the sample was stretched. There is a good interfacial adhesion between the fiber and the matrix, which improves the mechanical properties macroscopically.

However, when the short carbon fiber content reaches 35 wt%, it is observed that the fiber aggregation is remarkably severe. The interlayer gaps are also significantly enlarged, and the pores gradually increase in number. This will greatly reduce the interlayer bonding force, Moreover, a large number of fibers were pulled out, creating many holes and numerous bare carbon fibers with smooth surfaces. Therefore, it can be judged that the carbon fibers were pulled out rather than broken [14,34]. The smooth surface of the fibers also reflects that the interfacial adhesion between the short carbon fibers and the nylon matrix is weak [35]. The bubbles circled by the orange circles in Figure 9c2 were generated during the preparation of the SCF/PA composites [36]. As the short carbon fiber content increases, the number of bubbles gradually becomes more evident. Through Figure 9c1, it can be observed that due to the excessive increase in the number of fibers, nylon cannot fully blend with the short carbon fibers, resulting in a bundling phenomenon. It is precisely this bundling phenomenon of short carbon fibers, the gradual appearance of bubbles, and the increase in interlayer gaps that cause the nylon to be unable to transfer the force to more short carbon fibers when the specimen is under tension. Macroscopically, the mechanical properties of the short-carbon-fiber-reinforced nylon composites are reduced. This also reflects that with the increase in short carbon fibers, the interaction between the internal short carbon fibers and nylon becomes more obvious, causing the melt flow rate to decline, which is consistent with the previous research results. In addition, from Figure 9a1–c1, it can be seen that the short carbon fibers are basically aligned with the printing direction, which is beneficial to improving the mechanical properties of the printed specimens.

## 5. Conclusions

In this study, SCF/PA composites were prepared by using short carbon fibers and nylon through an extrusion process, and different contents of short-carbon-fiber-reinforced nylon composites were prepared the monofilament properties and printed sample properties were comprehensively evaluated and tested through experiments, and the sample after tensile testing was observed and analyzed in a cross-section by using a scanning electron microscope.

This paper investigated the effects of short-carbon-fiber (SCF) content on the mechanical properties and fluidity of SCF/PA composites. The results indicate that the melt flow rate decreases with the increase in the SCF content, and the melt flow index and its variation law are obtained. In terms of mechanical properties, it is not the case that the higher the SCF content is, the higher the tensile and flexural properties of the SCF/PA composites will be. Instead, the tensile strength and flexural strength reach their maximum values when the SCF content is 25 wt%. The tensile strength is 101.43 MPa, which is 17.01% higher than that of pure nylon. The flexural strength is 173.16 MPa, an increase of 21.4% compared to pure nylon, and the flexural modulus is 7546.99 MPa, 91.2% higher than that of pure nylon. The tensile modulus reaches its maximum value of 8124.84 MPa when the SCF content is 35 wt%, which is 125.37% higher than that of pure nylon. Observation of the fracture surface reveals that when the SCF content is 25 wt%, the carbon fibers are uniformly mixed with the resin, and the bonding ability is strong. However, for the 35 wt% SCF/PA composites with a high content, the increase in bubbles and fiber aggregation reduces the tensile and flexural strengths. The impact resistance increases with the increase in the carbon fiber content, but there is no linear relationship between the impact resistance and the SCF content. When the SCF content is 35 wt%, the impact resistance is optimal, reaching 6.02 KJ/m^2^, which is 38.1% higher than that of pure nylon, indicating a significant improvement in impact resistance. In the thermal deformation temperature experiment, the increase in the SCF content leads to a slight increase in the thermal deformation temperature, but the effect is limited. The research findings of this paper provide more basis and reference for different application scenarios of short-carbon-fiber-reinforced nylon composites.

## Figures and Tables

**Table 1:** Material performance indices.

	Tensile Strengthσ/GPa	Tensile ModulusE/GPa	$\;\mathrm{Density}\;\rho /g\cdot {cm}^{-3}$	Elongation Rate	DiameterD/μm	Melting Range °C	Glass Transition Temperature Tg/°C
nylon	0.1	3.8	1.2	/	/	230–240	85
short carbon fiber	3.30	225	1.82	1.7%	7	/	/

**Table 2:** 3D printing parameters.

	Printing TemperatureT/°C	Printing SpeedV/(mm/min)	Platform TemperatureT/°C	Floor HeightL/mm	Filling Density
parameters	300	100	80	0.2	100%

**Table 3:** Diameter test data.

	Carbon Fiber Content	1	2	3	4	5	Mean Value	Standard Deviation
diameter (mm)	0 wt%	1.77	1.75	1.748	1.76	1.765	1.7586	0.008
	15 wt%	1.762	1.74	1.738	1.748	1.76	1.7496	0.010
	25 wt%	1.74	1.74	1.743	1.747	1.747	1.7434	0.003
	35 wt%	1.75	1.74	1.742	1.741	1.744	1.7434	0.004

**Table 4:** Specific data on the tensile property of the monofilament.

		1	2	3	4	5	Mean Value	Standard Deviation
monofilament tensile strength (MPa)	PA	99.96	99.33	99.05	97.29	98.71	98.71	0.97
	15 wt%SCF/PA	173.92	165.88	170.54	185.63	168.59	172.91	6.88
	25 wt%SCF/PA	199.85	200.85	191.79	208.71	207.53	201.75	6.09
	35 wt%SCF/PA	162.87	159.76	166.5	171.96	162.57	164.73	4.2

**Table 5:** Tensile properties data.

Test Indicators		1	2	3	4	5	6	Average Values	Standard Deviation	Enhancement Range
tensile strength (MPa)	PA	86.40	86.36	86.62	87.3	86.89	86.22	86.63	0.37	\
	15%SCF/PA	93.42	94.84	95.54	93.34	91.82	94.98	93.99	1.3	7.8%
	25%SCF/PA	101.47	103.23	102.09	100.43	99.87	101.46	101.43	1.09	17.01%
	35%SCF/PA	91.72	90.95	91.33	91.33	91.48	91.86	91.45	0.29	5.56%
tensile modulus (MPa)	PA	3758.97	3569.02	3733.85	3380.01	3740.56	3448.67	3605.18	150.02	\
	15%SCF/PA	6445.64	7023.71	6591	6371.83	6751.97	6449.99	6605.69	223.94	83.23%
	25%SCF/PA	7500.52	7757.51	7540.41	7291.02	7543.56	7239.59	7478.77	172.2.54	107.45%
	35%SCF/PA	7998.84	8335.02	8086.34	8315.83	7896.38	8116.61	8124.84	158.26	125.37%
tensile strain (percentage)	PA	4.72	5.41	5	5.36	5.82	5.78	5.35	0.39	\
	15%SCF/PA	2.43	2.47	2.65	2.76	2.65	2.42	2.56	0.13	\
	25%SCF/PA	2.12	2.27	2.3	2.09	2.11	2.08	2.16	0.09	\
	35%SCF/PA	2.19	2.25	2.32	2.15	2.19	2.06	2.19	0.08	\

**Table 6:** Flexural properties data.

Test Indicators		1	2	3	4	5	6	Average Values	Standard Deviation	Enhancement Range
flexural strength (MPa)	PA	142.17	143.42	142.65	141.30	142.92	143.18	142.60	0.71	\
	15%SCF/PA	167.23	161.99	163.17	163.6	165.81	164.775	164.43	1.74	15.3%
	25%SCF/PA	172.96	175.23	174.55	173.63	171.19	171.4	173.16	1.5	21.4%
	35%SCF/PA	159.16	158.15	158.79	154.32	155.36	155.14	156.82	1.9	10%
flexural modulus (MPa)	PA	3941.25	3910.98	3992.72	3909.28	3975.97	3952.52	3947.12	30.86	\
	15%SCF/PA	6507.4	6615.31	6416.08	6546.28	6556.01	6387.63	6504.79	79.75	64.80%
	25%SCF/PA	7574.41	7685.3	7494.85	7603.46	7510.69	7413.25	7546.99	86.6	91.2%
	35%SCF/PA	7374.83	7392.22	7235.28	7290.51	7300.49	7324.25	7319.60	52.71	85.44%

**Table 7:** Impact test data of short-carbon-fiber composites.

	Materials	1	2	3	4	5	6	Average Value	Standard Deviation	Enhancement Range
impact strength (KJ/m^2^)	PA	4.68	4.45	4.43	4.02	4.51	4.04	4.36	0.24	\
	15 wt%SCF/PA	5.11	5.12	4.71	4.89	4.99	5.14	4.99	0.15	14.4%
	25 wt%SCF/PA	5.48	5.31	5.5	5.21	5.36	5.2	5.34	0.12	22.5%
	35 wt%SCF/PA	6.47	5.66	5.99	5.66	6.15	6.16	6.02	0.29	38.1%

**Table 8:** Heat distortion temperature test data.

	Sample 1	Sample 2	Sample 3	Average Value	Standard Deviation
PA	74.9	75.9	75.1	78.1	0.43
15 wt%SCF/PA	80.8	81.4	80.6	80.9	0.34
25 wt%SCF/PA	81.2	81.8	80.6	81.4	0.26
35 wt%SCF/PA	82.2	82.8	82.2	82.4	0.28

## Data Availability

All relevant data are within the paper.
